# Cardiovascular medication use and risks of colon cancer recurrences and additional cancer events: a cohort study

**DOI:** 10.1186/s12885-019-5493-8

**Published:** 2019-03-27

**Authors:** Erin J. A. Bowles, Onchee Yu, Rebecca Ziebell, Lu Chen, Denise M. Boudreau, Debra P. Ritzwoller, Rebecca A. Hubbard, Jennifer M. Boggs, Andrea N. Burnett-Hartman, Andrew Sterrett, Monica Fujii, Jessica Chubak

**Affiliations:** 1 Kaiser Permanente Washington Health Research Institute, 1730 Minor Avenue, Suite 1600, Seattle, WA 98101 USA; 2 School of Pharmacy, University of Washington, Seattle, Washington 98195 USA; 3 Department of Epidemiology, School of Public Health, University of Washington, Seattle, Washington USA; 4 Kaiser Permanente Colorado Institute for Health Research, 2550 S Parker Rd Suite 200, Aurora, Colorado 80014 USA; 5 Department of Biostatistics, Epidemiology, and Informatics, Perelman School of Medicine, University of Pennsylvania, 604 Blockley Hall, 423 Guardian Drive, Philadelphia, PA 19104 USA

**Keywords:** Colon cancer, Statin, Antihypertensive, Recurrence

## Abstract

**Background:**

Cardiovascular medications may be associated with cancer development, but little is known about their association with cancer recurrence. Medications such as statins and antihypertensives may be commonly used among colon cancer survivors, who are, on average, diagnosed in their mid-60s. We described the associations between statins and antihypertensive medications and colon cancer recurrence in a large, population-based study.

**Methods:**

We conducted a cohort study among adults with stage I-IIIA colon cancer diagnosed in 1995–2014 in two Kaiser Permanente regions, Colorado and Washington. Statin and antihypertensive use were obtained from electronic pharmacy dispensing data. People were classified as medication users on the date of their first dispensing after cohort entry, which started 90 days after completing cancer treatment, continuing through the earliest of death, health plan disenrollment, or chart abstraction. We collected outcome information from medical record abstraction and tumor registries on colon cancer recurrences and second primary cancers. Using Cox proportional hazards multivariable models, we estimated hazard ratios (HRs) with 95% confidence intervals (CIs) for colon cancer recurrences and any cancer event (recurrences and new primaries at any anatomic site) comparing medication users to non-users.

**Results:**

Among 2039 people, 937 (46%) used statins and 1425 (70%) used antihypertensives at any point during a median of 4.9 years of follow-up; 460 people had any additional cancer event, including 152 with a colon cancer recurrence. Statin use was not associated with colon cancer recurrence (HR = 1.09, 95%CI = 0.65–1.85) or any cancer event (HR = 1.12, 95%CI = 0.85–1.47), nor was antihypertensive use associated with recurrence (HR = 0.73, 95%CI = 0.44–1.21) or any cancer event (HR = 0.93, 95%CI = 0.70–1.24).

**Conclusions:**

Our results suggest no association between cardiovascular medication use and the risk of recurrence or any additional cancer, and may provide reassurance to colon cancer survivors.

## Background

Colorectal cancer is the third most commonly diagnosed cancer in men and women [1] with an estimated 135,430 new cancers diagnosed in the United States in 2017 [2]. When diagnosed at an early stage, prognosis is excellent with nearly 90% of patients with local disease and 71% of patients with regional disease surviving at least 5 years after diagnosis [2]. With a median age at diagnosis of 67 [2], cancer survivors often deal with comorbidities, such as cardiovascular disease, that need treatment in addition to their cancer. These diseases and their treatments may or may not impact their risk of recurrence and overall mortality.

Approximately 20% of the U.S. adult population uses statins to reduce high cholesterol, a major risk factor for heart disease [3]. Statins block the HMG-CoA reductase enzyme in the liver, which is a rate-controlling enzyme in the production of cholesterol, thereby lowering cholesterol levels and reducing a person’s risk of coronary heart disease [4]. However, statins may have other beneficial effects, particularly when it comes to cancer. Because statins decrease cell proliferation and increase apoptosis, there is a mechanism to suggest they may decrease risks of incident cancer and cancer recurrence [5–10], and potentially even cancer deaths [11–19]. Three studies examined the impact of statins on colon cancer recurrence [14, 18, 20], but none found a reduced risk of recurrence among statin users compared to non-users. Two of these studies were large, population-based analyses conducted outside the United States while the third included only stage III colorectal cancer patients. We are unaware of any U.S.-based study that has evaluated the association between statin use and cancer recurrence in early-stage colon cancer patients.

Antihypertensives are another commonly used medication in the U.S.; these medications treat high blood pressure (or hypertension), which affects 1 in 3 U.S. adults [21]. Certain types of commonly used antihypertensive, including angiotensin-converting enzyme inhibitors (ACEIs), angiotensin-II receptor blockers (ARBs), and beta blockers (BBs), may limit tumor cell growth and replication, which is hypothesized to reduce recurrence risk [22–26]. Three epidemiologic studies [27–29] investigated this hypothesis and found mixed results. Calcium channel blockers (CCBs) may inhibit apoptosis while thiazide diuretics (TDs) can increase insulin resistance (an established risk factor for colon cancer) [30, 31]; therefore, CCBs and diuretics are hypothesized to increase risk for colon cancer and progression [32, 33]. We are not aware of any epidemiological studies that have examined this latter hypothesis.

Statin and antihypertensive use is common among colon cancer survivors. Therefore, it is important to understand the long-term risks and benefits of these drugs in this population. With data on medication use from electronic pharmacy databases and recurrence outcomes from manual medical record review, we evaluated the associations between statin and antihypertensive use and colon cancer recurrence in a large, population-based study from the United States.

## Methods

RECORD (**Re**currence of **Co**lon cancer in **R**elation to **D**rug use) is a cohort study within the Health Care System Research Network’s (HCSRN) [34] Cancer Research Network [35]. The overall goal of RECORD is to examine commonly used medication exposures in relation to colon cancer and recurrence outcomes among adults with incident colon cancer diagnosed in two health care systems: Kaiser Permanente Washington (KPWA) and Kaiser Permanente Colorado (KPCO). The study received human subjects approval from Kaiser Permanente Washington Health Research Institute Human Subjects Review Committee (to which KPCO ceded) with a waiver of consent.

### Study setting and population

Eligible study participants were > 18 years of age at the time of diagnosis of an incident American Joint Committee on Cancer 6th ed. (AJCC) stage I-IIIA malignant adenocarcinoma in the colon (including the appendix and rectosigmoid junction, excluding the rectum) from 1995 through 2014 (*N* = 3326). People with colon cancer were identified through the HCSRN’s Virtual Data Warehouse (VDW) [36]. The VDW is a decentralized data model with mutually agreed upon variable definitions across HCSRN sites; this standardization of automated health plan data allows for efficient cross-site collaboration. Both KPWA and KPCO VDWs contain a Tumor table, which is populated by incident cancer cases from the local Seattle-Puget Sound Surveillance Epidemiology and End Results (SEER) registry (KPWA) or from the health plan’s internal registry (KPCO). We collected data on all study subjects starting 12 months before diagnosis through the end of follow-up. Study follow-up (i.e., cohort entry) began 90 days after the end of the primary treatment (surgery, chemotherapy, and/or radiation). End of follow-up occurred at the earliest of death, disenrollment from health plan, or date of chart abstraction (through August 30, 2016).

**Fig. 1:**
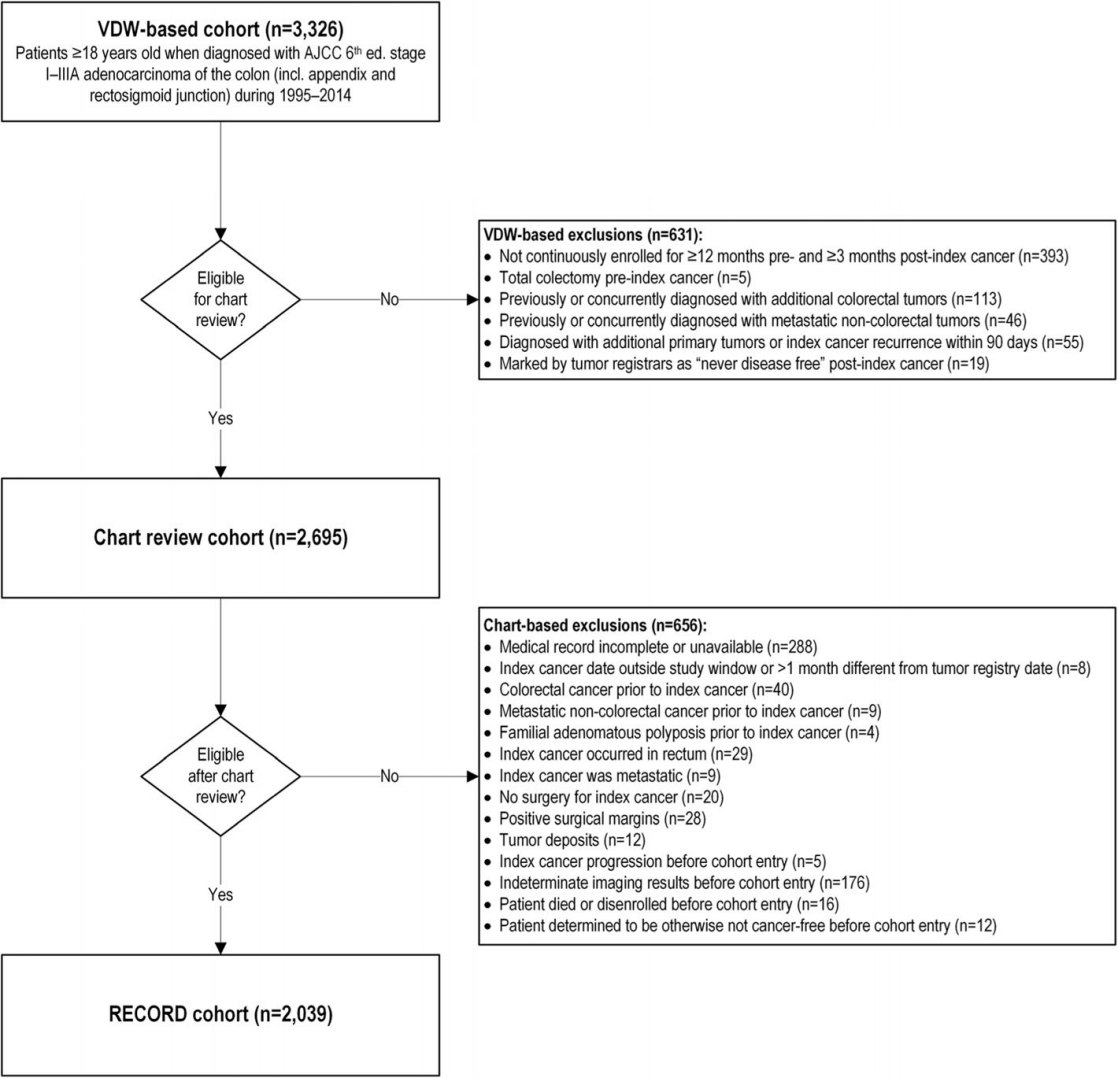
Inclusion and exclusion criteria for RECORD cohort study. This figure shows the number of people included in the RECORD study with Ns for each exclusion criteria

We excluded 631 patients from our sample because they did not meet certain eligibility criteria (see Fig. 1 for detailed criteria). Among 2695 people who were eligible for medical record review, we excluded an additional 656 patients because they had no medical record available, had a prior colorectal cancer diagnosis or metastatic cancer at another site, or had findings suggestive of cancer progression within 90 days after cancer treatment completion. A total of 2039 people remained eligible for analyses after chart abstraction.

### Medication use

We identified use of statins and antihypertensive from outpatient pharmacy dispensings in the VDW Pharmacy. All medication classes, brand names, and generic names were reviewed by a pharmacist (DMB) for inclusion and exclusion. For statins, we evaluated all statins combined and the three most common types of statins in our population separately (atorvastatin, lovastatin, and simvastatin). Any other form of statin use was grouped together as “other statins.” We excluded non-statin lipid-lowering drugs because these were prescribed rarely. For antihypertensive, we evaluated all antihypertensive combined and each class of antihypertensive separately (ACEI, ARB, BB, CCB, and TD).

We determined whether people used any medications of interest in the 12 months before incident colon cancer diagnosis. From cohort entry through end of follow-up, we collected information on individual pharmacy dispensing’s so that we could evaluate medication exposure in a time-varying manner. People who were already using a medication at cohort entry were considered exposed and remained that way through the end of follow-up. People who were not using the medication at cohort entry were considered unexposed starting at cohort entry until they started using the drug. People became users on the date of their first prescription after cohort entry and remained classified as exposed through the end of follow-up. We treated exposures in this manner because we were interested in evaluating the risk of recurrence associated with any exposure to these medications after cohort entry.

### Outcomes

Our primary outcome was colon cancer recurrence. Recurrences were collected via medical record abstraction at KPWA and via the tumor registry at KPCO. At both study sites, recurrence was defined by a clinical diagnosis in the medical record. Both sites collected date of recurrence, consistency of recurrence histology with index cancer (yes/no), and recurrence location (local = anastomosis or site of primary tumor or incision or elsewhere in the colon including appendix or rectum; regional = in nearby nodes or on the outside of adjacent organs; distant = distant nodes, peritoneum, or inside other organs; or unknown). Information on death and disenrollment was obtained from the VDW.

Our second outcome of interest was any cancer event, defined by a colon cancer recurrence, second primary colon cancer, recurrence of another cancer, or new primary cancer at any site. Medical record abstractors at both sites collected data on recurrences of other cancers and supplemented tumor registry data on second primary colorectal cancer diagnoses. Post-hoc, we manually re-reviewed charts of persons who died of colon cancer without a documented recurrence or second primary colon cancer to ensure that no study outcomes were missed. Because many of these cases had a cancer of unknown type shortly before death, we included these events in a broader definition of recurrence in sensitivity analyses.

### Covariates

We collected information on covariates from the VDW and medical record abstraction. From the VDW, we extracted cancer data on diagnosis age, diagnosis date, stage, and primary treatment. We supplemented treatment data through medical record abstraction. We extracted information on sex, race, ethnicity, and comorbid conditions (Charlson score [37], hypertension diagnosis, high cholesterol diagnosis, diabetes diagnosis) from the VDW Diagnosis tables in the year before cancer diagnosis and during follow-up. Statin and antihypertensive use in the year before diagnosis were extracted from the VDW whereas aspirin use and smoking history were abstracted from medical records. Body mass index was ascertained from the VDW in the year before cancer diagnosis and during follow-up with missing information supplemented by medical record abstraction.

### Statistical analyses

We calculated unadjusted incidence rates per 1000 person-years for recurrences and any cancer events among medication users and non-users starting at cohort entry through the end of follow-up. We used Cox proportional hazards models to estimate hazard ratios (HR) with 95% confidence intervals (CIs) for the associations between medication use and risks of 1) colon cancer recurrence, and 2) any cancer event. The exposure window for statins and antihypertensive started at cohort entry. Once a person was exposed to these medications, they remained exposed through the end of follow-up (unidirectional time-varying exposure). Study subjects were censored at the earliest of death, health plan disenrollment, or chart abstraction date. In the recurrence analysis, we also censored subjects at the diagnosis of a second primary cancer at any site (including colorectal) or recurrence of a non-colon cancer. Statin and antihypertensive exposures were included in the same model and mutually adjusted for one another. For the statins analysis, the reference group was people who did not use any statin medication. For the antihypertensive analysis, the reference group was people who did not use any antihypertensive medication. For analyses looking at specific types or classes of statins or antihypertensive, the reference groups were people who did not use that specific type or class of medication.

We conducted analyses using three sets of models: 1) minimally adjusted models, adjusting for age and diagnosis year (both using natural cubic splines with knots at tertiles), sex, stage at diagnosis, and study site; 2) fully adjusted models, including all covariates in the minimally adjusted model plus race (black, white, other/unknown), smoking (time-varying coded as ever, never), BMI (categories measured in the year before diagnosis), Charlson comorbidity score (from the year before diagnosis coded as 0, 1, 2, 3+, unknown), and any statin and/or antihypertensive use in the year before diagnosis; and 3) sensitivity models, including all covariates in the fully adjusted model plus diagnoses in the year before colon cancer diagnosis (diabetes, hypertension, and hypercholesterolemia), aspirin use in the year before diagnosis, chemotherapy treatment, and radiation therapy. We only present results from the fully adjusted models because results from all models were similar.

We tested the proportional hazards assumption in the fully adjusted models of our outcomes (recurrence and any cancer) by including interaction terms between statin use and the log of analysis time, and antihypertensive use and the log of analysis time. The proportional hazards assumption was satisfied for all analyses except the model for antihypertensive use and any cancer outcome. Thus, we visually examined stratified cumulative hazard plots over time to determine if hazards were approximately proportional within discrete intervals of follow-up time. Visual examination of the stratified cumulative hazard plots and Schoenfeld residuals showed very little deviation over time. Therefore, we report a single HR over the entire study period.

We used a *p*-value < 0.05 to define statistical significance. All analyses were conducted in SAS version 9.4 (SAS Institute, Cary, North Carolina).

## Results

Among 2039 colon cancer survivors, 937 (46.0%) used statins after cohort entry (Table 1). Compared to non-users, a greater proportion of statin users were male (53% vs 43% among non-users), ever smokers (60% vs 51%), and had a greater comorbidity burden at diagnosis (29% vs 13% with Charlson score > 2, 33% vs 8% with diabetes, 53% vs 14% with high cholesterol, and 67% vs 44% with hypertension). There were no major differences in colon cancer tumor characteristics between statin users and non-users. A little more than half (54%) of statin users after cohort entry had also used statins in the year before colon cancer diagnosis. A larger proportion of statin users used aspirin (44%) or antihypertensive (68%) in the year before diagnosis compared to non-users of statins (26 and 40%, respectively). After cohort entry, the most common type of statin used across all statin users was simvastatin (73%), followed by lovastatin (45%) and atorvastatin (24%).

**Table 1:** Population characteristics stratified by any statin use after cohort entry through end of follow-up

	All (*n* = 2039)		No statin use (*n* = 1102)		Statin use any time after cohort entry (*n* = 937)	
	n	(%)	n	(%)	n	(%)
Year of colon cancer diagnosis						
1995–1999	410	(20.1)	264	(24.0)	146	(15.6)
2000–2004	569	(27.9)	302	(27.4)	267	(28.5)
2005–2009	579	(28.4)	275	(25.0)	304	(32.4)
2010–2014	481	(23.6)	261	(23.7)	220	(23.5)
Age at diagnosis (years)						
mean (SD)	69.9	(11.6)	69.6	(12.7)	70.2	(10.1)
< 50	94	(4.6)	73	(6.6)	21	(2.2)
50–59	294	(14.4)	167	(15.2)	127	(13.6)
60–69	530	(26.0)	260	(23.6)	270	(28.8)
70–79	677	(33.2)	327	(29.7)	350	(37.4)
80+	444	(21.8)	275	(25.0)	169	(18.0)
Sex						
Female	1066	(52.3)	626	(56.8)	440	(47.0)
Male	973	(47.7)	476	(43.2)	497	(53.0)
Hispanic ethnicity						
Not Hispanic	1742	(85.4)	916	(83.1)	826	(88.2)
Hispanic	93	(4.6)	42	(3.8)	51	(5.4)
Unknown^a^	204	(10.0)	144	(13.1)	60	(6.4)
Race						
White	1582	(77.6)	844	(76.6)	738	(78.8)
Black	70	(3.4)	32	(2.9)	38	(4.1)
Asian	66	(3.2)	36	(3.3)	30	(3.2)
American Indian/Alaska Native	9	(0.4)	3	(0.3)	6	(0.6)
Hawaiian/Pacific Islander	5	(0.2)	3	(0.3)	2	(0.2)
Multiple race	19	(0.9)	9	(0.8)	10	(1.1)
Other/Unknown	288	(14.1)	175	(15.9)	113	(12.1)
Smoking before diagnosis						
Never	916	(45.2)	538	(49.1)	378	(40.5)
Ever	1112	(54.8)	557	(50.9)	555	(59.5)
Unknown	11		7		4	
Charlson score at diagnosis						
mean (SD)	0.8	(1.4)	0.6	(1.0)	1.2	(1.6)
0	1085	(53.2)	688	(62.4)	397	(42.4)
1	429	(21.0)	207	(18.8)	222	(23.7)
2	184	(9.0)	75	(6.8)	109	(11.6)
3+	225	(11.0)	65	(5.9)	160	(17.1)
Unknown^a^	116	(5.7)	67	(6.1)	49	(5.2)
BMI at diagnosis (kg/m^2^)						
mean (SD)	27.9	(5.9)	27	(5.6)	29	(6.1)
Underweight (< 18.5)	41	(2.1)	29	(2.8)	12	(1.3)
Normal (18.5–24.9)	610	(31.4)	386	(37.9)	224	(24.3)
Overweight (25–29.9)	702	(36.2)	351	(34.5)	351	(38.0)
Obese (30–34.9)	380	(19.6)	159	(15.6)	221	(23.9)
Morbidly obese (35+)	208	(10.7)	93	(9.1)	115	(12.5)
Unknown	98		84		14	
Diagnoses in the year before colon cancer						
Diabetes	400	(19.6)	93	(8.4)	307	(32.8)
Hyperlipidemia/hypercholesterolemia	655	(32.1)	159	(14.4)	496	(52.9)
Hypertension	1111	(54.5)	481	(43.6)	630	(67.2)
Stage at colon cancer diagnosis						
I	911	(44.7)	457	(41.5)	454	(48.5)
IIA	935	(45.9)	523	(47.5)	412	(44.0)
IIB	117	(5.7)	78	(7.1)	39	(4.2)
IIIA	76	(3.7)	44	(4.0)	32	(3.4)
Grade						
Grade I	187	(9.7)	89	(8.5)	98	(11.1)
Grade II	1423	(73.8)	784	(75.0)	639	(72.4)
Grade III	294	(15.2)	158	(15.1)	136	(15.4)
Grade IV	24	(1.2)	14	(1.3)	10	(1.1)
Unknown, not stated, or N/A	111		57		54	
Cancer treatment received						
Chemotherapy	277	(13.6)	170	(15.4)	107	(11.4)
Radiation	30	(1.5)	15	(1.4)	15	(1.6)
Medication use in the year before colon cancer diagnosis^b^						
Any Statin	542	(26.6)	34	(3.1)	508	(54.2)
Atorvastatin	29	(1.4)	1	(0.1)	28	(3.0)
Lovastatin	235	(11.5)	13	(1.2)	222	(23.7)
Simvastatin	306	(15.0)	20	(1.8)	286	(30.5)
Other statin	22	(1.1)	3	(0.3)	19	(2.0)
Aspirin	685	(34.1)	280	(25.9)	405	(43.8)
Any antihypertensive	1078	(52.9)	444	(40.3)	634	(67.7)
Common types of statin use after colon cancer diagnosis^b^						
Atorvastatin	220	(10.8)	0	(0)	220	(23.5)
Lovastatin	425	(20.8)	0	(0)	425	(45.4)
Simvastatin	683	(33.5)	0	(0)	683	(72.9)
Other statin	101	(5.0)	0	(0)	101	(10.8)

^a^Unknown categories for race and Charlson score are included in percent calculations because they are included in the multivariable models

^b^Medication categories not mutually exclusive because patients could have used more than one type of medication before diagnosis and during study follow-up

Antihypertensive users (*N* = 1425, 69.9% of study population) were older than non-users (25% over 80 years vs. 15% among non-users), with a larger proportion of ever smokers (57% vs 50%) and a greater comorbidity burden at diagnosis (26% vs 7% with Charlson score > 2, 26% vs 6% with a previous diabetes diagnosis, and 71% vs 17% with a previous hypertension diagnosis) (Table 2). Most antihypertensive users after cohort entry also used antihypertensive in the year before diagnosis (72%). A larger proportion of antihypertensive users used aspirin (40%) or statins (34%) in the year before colon cancer diagnosis compared to non-users (20 and 9%, respectively). After cohort entry, the most common types of antihypertensive dispensed were BBs (63%) and ACEIs (62%), followed by TDs (50%), CCBs (39%), and ARBs (16%).

**Table 2:** Population characteristics stratified by any antihypertensive use after cohort entry through end of follow-up

	All (*n* = 2039)		No antihypertensive use (*n* = 614)		Any antihypertensive use (*n* = 1425)	
	n	(%)	n	(%)	n	(%)
Year of colon cancer diagnosis						
1995–1999	410	(20.1)	105	(17.1)	305	(21.4)
2000–2004	569	(27.9)	157	(25.6)	412	(28.9)
2005–2009	579	(28.4)	171	(27.9)	408	(28.6)
2010–2014	481	(23.6)	181	(29.5)	300	(21.1)
Age at diagnosis (years)						
mean (SD)	69.9	(11.6)	65.8	(12.7)	71.6	(10.6)
< 50	94	(4.6)	59	(9.6)	35	(2.5)
50–59	294	(14.4)	136	(22.1)	158	(11.1)
60–69	530	(26.0)	165	(26.9)	365	(25.6)
70–79	677	(33.2)	162	(26.4)	515	(36.1)
80+	444	(21.8)	92	(15.0)	352	(24.7)
Sex						
Female	1066	(52.3)	323	(52.6)	743	(52.1)
Male	973	(47.7)	291	(47.4)	682	(47.9)
Hispanic ethnicity						
Not Hispanic	1742	(85.4)	513	(83.6)	1229	(86.2)
Hispanic	93	(4.6)	29	(4.7)	64	(4.5)
Unknown^a^	204	(10.0)	72	(11.7)	132	(9.3)
Race						
White	1582	(77.6)	467	(76.1)	1115	(78.2)
Black	70	(3.4)	15	(2.4)	55	(3.9)
Asian	66	(3.2)	23	(3.7)	43	(3.0)
American Indian/Alaska Native	9	(0.4)	1	(0.2)	8	(0.6)
Hawaiian/Pacific Islander	5	(0.2)	2	(0.3)	3	(0.2)
Multiple race	19	(0.9)	7	(1.1)	12	(0.8)
Other/Unknown	288	(14.1)	99	(16.1)	189	(13.3)
Smoking before diagnosis						
Never	916	(45.2)	307	(50.4)	609	(42.9)
Ever	1112	(54.8)	302	(49.6)	810	(57.1)
Unknown	11		5		6	
Charlson score at diagnosis						
mean (SD)	0.8	(1.4)	0.3	(0.8)	1.1	(1.5)
0	1085	(53.2)	453	(73.8)	632	(44.4)
1	429	(21.0)	87	(14.1)	342	(24.0)
2	184	(9.0)	19	(3.1)	165	(11.6)
3+	225	(11.0)	22	(3.6)	203	(14.3)
Unknown^a^	116	(5.7)	33	(5.4)	83	(5.8)
BMI at diagnosis (kg/m^2^)						
mean (SD)	27.9	(5.9)	26.7	(5.3)	28.5	(6.1)
Underweight (< 18.5)	41	(2.1)	17	(2.9)	24	(1.8)
Normal (18.5–24.9)	610	(31.4)	223	(38.4)	387	(28.4)
Overweight (25–29.9)	702	(36.2)	212	(36.6)	490	(36.0)
Obese (30–34.9)	380	(19.6)	90	(15.5)	290	(21.3)
Morbidly obese (35+)	208	(10.7)	38	(6.6)	170	(12.5)
Unknown	98		34		64	
Diagnoses in the year before colon cancer						
Diabetes	400	(19.6)	36	(5.9)	364	(25.5)
Hyperlipidemia/hypercholesterolemia	655	(32.1)	128	(20.8)	527	(37.0)
Hypertension	1111	(54.5)	106	(17.3)	1005	(70.5)
Stage at colon cancer diagnosis						
I	911	(44.7)	286	(46.6)	625	(43.9)
IIA	935	(45.9)	247	(40.2)	688	(48.3)
IIB	117	(5.7)	52	(8.5)	65	(4.6)
IIIA	76	(3.7)	29	(4.7)	47	(3.3)
Grade						
Grade I	187	(9.7)	48	(8.4)	139	(10.3)
Grade II	1423	(73.8)	434	(75.6)	989	(73.0)
Grade III	294	(15.2)	84	(14.6)	210	(15.5)
Grade IV	24	(1.2)	8	(1.4)	16	(1.2)
Unknown, not stated, or N/A	111		40		71	
Cancer treatment received						
Chemotherapy	277	(13.6)	112	(18.2)	165	(11.6)
Radiation	30	(1.5)	11	(1.8)	19	(1.3)
Medication use in the year before colon cancer diagnosis^b^						
Any Statin	542	(26.6)	56	(9.1)	486	(34.1)
Aspirin	685	(34.1)	120	(19.9)	565	(40.2)
Any antihypertensive	1078	(52.9)	51	(8.3)	1027	(72.1)
ACE inhibitor	524	(25.7)	15	(2.4)	509	(35.7)
Angiotensin receptor blocker	92	(4.5)	1	(0.2)	91	(6.4)
Beta blocker	528	(25.9)	16	(2.6)	512	(35.9)
Calcium channel blocker	287	(14.1)	10	(1.6)	277	(19.4)
Diuretic	479	(23.5)	20	(3.3)	459	(32.2)
Type of antihypertensive after colon cancer diagnosis^b^						
ACE inhibitor	878	(43.1)	0	(0)	878	(61.6)
Angiotensin receptor blocker	226	(11.1)	0	(0)	226	(15.9)
Beta blocker	890	(43.6)	0	(0)	890	(62.5)
Calcium channel blocker	559	(27.4)	0	(0)	559	(39.2)
Diuretic	713	(35.0)	0	(0)	713	(50.0)

^a^Unknown categories for race and Charlson score are included in percent calculations because they are included in the multivariable models

^b^Medication categories not mutually exclusive because patients could have used more than one type of medication before diagnosis and during study follow-up

A total of 152 people experienced a colon cancer recurrence (including 118 distant recurrences), and 460 had any cancer event (Table 3). Among statin users, the unadjusted incidence of recurrence was 10.4 per 1000 person-years (95%CI = 7.7–13.7) compared to 14.3 per 1000 person-years (95%CI = 11.6–17.3) among people who did not use statins (Table 3). There was no association between any statin use and risk of recurrence after confounder adjustment (HR = 1.09, 95%CI = 0.65–1.85), or between different types of statins and risk of recurrence (see Table 3 for individual HRs). The adjusted associations between overall statin use or types of statins and risk of any cancer event were also not significant.

**Table 3:** Risks of colon cancer recurrences and any additional cancers associated with statin use

			Recurrence			Any cancer event		
	N at risk^a^	Person-years	N	Crude incidence rate per 1000 person-years (95%CI)	Fully adjusted recurrence HR (95%CI)^b^	N	Crude incidence rate per 1000 person-years (95%CI)	Fully adjusted any cancer HR (95% CI)^b^
Any statin								
No statin use	1605	7222	103	14.3 (11.6–17.3)	1 (ref)	273	37.8 (33.4–42.6)	1 (ref)
Statin use	937	4734	49	10.4 (7.7–13.7)	1.09 (0.65–1.85)	187	39.5 (34.0–45.6)	1.12 (0.85–1.47)
By type of statin^c^								
Atorvastatin use (ref = no atorvastatin use)	219	541	6	11.1 (4.1–24.1)	1.56 (0.63–3.87)	22	40.7 (25.5–61.6)	0.94 (0.59–1.49)
Lovastatin use (ref = no lovastatin use)	425	2685	28	10.4 (6.9–15.1)	1.47 (0.89–2.43)	107	39.9 (32.7–48.2)	1.27 (0.98–1.65)
Simvastatin use (ref = no simvastatin use)	683	3335	30	9.0 (6.1–12.8)	0.94 (0.56–1.57)	130	39.0 (32.6–46.3)	1.03 (0.79–1.34)
Other statin use (ref = no other statin use)	101	329	1	3.0 (0.1–16.9)	0.28 (0.04–2.14)	17	51.6 (30.1–82.6)	1.44 (0.86–2.41)

^a^N at risk differs from N in Table 1 because statin exposures are time-varying. Therefore, participants contribute unexposed time until the day they meet exposure criteria

^b^Fully adjusted model included: age and diagnosis year (both using natural cubic splines with knots at tertiles), sex (male/female), stage at diagnosis (I/IIA/IIB/IIIA), study site (KPWA/KPCO), race (white/black/other & unknown), time-varying smoking (yes/no), BMI at diagnosis (< 25.0/25.0–29.9/30.0+ kg/m^2^), Charlson comorbidity score in the year before diagnosis (0/1/2/3+), statin use in the year before diagnosis (yes/no), antihypertensive use in the year before diagnosis (yes/no), and time-varying antihypertensive use after cohort entry (yes/no)

^c^Each statin exposure adjusted for all other statin exposures. People could be exposed to multiple types of statins during the study follow-up

Abbreviations: *HR* hazard ratio, *CI* confidence interval

Among antihypertensive users, the unadjusted incidence of recurrence was 11.3 per 1000 person-years (95%CI = 9.0–13.9) compared to 15.5 per 1000 person-years (95%CI = 11.9–19.8) among nonusers of antihypertensive medications (Table 4). There was no association between any antihypertensive use and risk of recurrence after adjustment (HR = 0.73, 95%CI = 0.44–1.21), or between individual antihypertensive classes and recurrence (see Table 4 for individual HRs). There were no statistically significant associations between any antihypertensive use or individual antihypertensive classes and risk of any cancer event.

**Table 4:** Risks of colon cancer recurrences and any additional cancers associated with antihypertensive use

			Recurrence			Any cancer event		
	N at risk^a^	person-years	N	Crude incidence rate per 1000 person-years (95%CI)	Fully adjusted recurrence HR (95%CI)^b^	N	Crude incidence rate per 1000 person-years (95%CI)	Fully adjusted any cancer HR (95%CI)^b^
Any antihypertensive medication								
No antihypertensive use	1083	4134	64	15.5 (11.9–19.8)	1 (ref)	155	37.5 (31.8–43.9)	1 (ref)
Antihypertensive use	1425	7823	88	11.3 (9.0–13.9)	0.73 (0.44–1.21)	305	39.0 (34.7–43.6)	0.94 (0.70–1.24)
By type of antihypertensive^c^								
ACEI use (ref = no ACEI use)	878	4546	43	9.5 (6.8–12.7)	0.84 (0.55–1.28)	168	37.0 (31.6–43.0)	0.93 (0.74–1.17)
ARB use (ref = no ARB use)	226	1042	10	9.6 (4.6–17.6)	1.17 (0.58–2.36)	42	40.3 (29.0–54.5)	1.06 (0.75–1.49)
BB use (ref = no BB use)	890	4734	44	9.3 (6.8–12.5)	0.76 (0.50–1.14)	177	37.4 (32.1–43.3)	0.90 (0.72–1.12)
CCB use (ref = no CCB use)	559	2720	20	7.4 (4.5–11.4)	0.62 (0.37–1.04)	100	36.8 (29.9–44.7)	0.92 (0.71–1.19)
TD use (Ref = no TD use)	713	4191	37	8.8 (6.2–12.2)	0.75 (0.49–1.17)	137	32.7 (27.4–38.6)	0.80 (0.63–1.01)

^a^N at risk differs from N in Table 2 because antihypertensive exposures are time-varying. Therefore, participants contribute unexposed time until the day they meet exposure criteria

^b^Fully adjusted model included: age and diagnosis year (both using natural cubic splines with knots at tertiles), sex (male/female), stage at diagnosis (I/IIA/IIB/IIIA), study site (KPWA/KPCO), race (white/black/other & unknown), time-varying smoking (yes/no), BMI at diagnosis (< 25.0/25.0–29.9/30.0+ kg/m^2^), Charlson comorbidity score in the year before diagnosis (0/1/2/3+), statin use in the year before diagnosis (yes/no), antihypertensive use in the year before diagnosis (yes/no), and time-varying statin use after cohort entry (yes/no)

^c^Each antihypertensive exposure adjusted for all other antihypertensive exposures. People could be exposed to multiple types of antihypertensives during the study follow-up

Abbreviations: *HR* hazard ratio, *CI* confidence interval, *ACE* angiotensin-converting enzyme inhibitor, *ARB* angiotensin-II receptor blockers, *BB* beta blocker, *CCB* calcium channel blocker, *TD* thiazide diuretic

Sensitivity analyses included cancers of unknown type as recurrences and adjusted for additional baseline covariates. These analyses did not change our results and are not presented.

## Discussion

Although previous studies have shown that statin and antihypertensive medications are associated with colon cancer risk, and there are known mechanisms to support these associations, we saw no significant associations between these medications and risks of colon cancer recurrence or any cancer event. Many older adults use one or both medications to manage cardiovascular disease. Our results should reassure patients and their providers that these medications do not appear to impact recurrence risk in colon cancer survivors.

Our null results for statin use are similar to those found in three prior studies [14, 18, 20]. In a large study by Lash et al. (*N* = 21,152) which used data from population-based Danish medical registries including time-varying statin prescription information, the HR for recurrence was 1.01 (95%CI = 0.93–1.09) and the HR for colorectal cancer specific mortality was 0.72 (95%CI = 0.66–0.79) [18]. The distribution of recurrence sites was similar among statin users and non-users suggesting the reduced risk of death were not due to cancer or recurrence. Several additional observational studies have looked at the associations between statin use and colorectal cancer-specific survival and found significantly reduced risks of colon cancer mortality or trends suggesting better survival among statin users compared to non-users [11–13, 16, 19]. We did not evaluate mortality as an outcome in our study because of the potential for confounding by indication (i.e., the reason the person started taking a drug, such as a comorbid disease, is also associated with mortality). However, the evidence that statins may reduce cancer-specific mortality should be considered alongside evidence from a recent study suggesting the observed associations between statins and reduced colon cancer mortality were due to selection and immortal time biases [38].

Previous studies evaluating antihypertensive use and colon cancer recurrence risk have produced mixed results. The largest study, by Jansen et al., was conducted among 1820 colon cancer survivors in Germany. After obtaining self-reported information on prior BB use, the study found no association with recurrence (HR = 1.04, 95%CI = 0.79–1.38) [27]. Two smaller studies were conducted in the Veterans Administration in Texas, which includes a predominately male population, and collected information on antihypertensive used from automated dispensing data. One (*N* = 262) found a reduced risk of tumor progression among people with stage III or IV colon cancer who used ACEIs, ARBs, or BBs (HR = 0.59, 95%CI = 0.36–0.99), though the HRs for the individual drug classes were not statistically significant [28]. The other (*N* = 55) noted a significantly increased risk for tumor metastases among stage II colon cancer survivors who used ACEIs (HR = 4.5, *p* = 0.007, CI not reported) [29].

Our study was larger than any of these prior three analyses, relied on electronic, longitudinal medication dispensing data (rather than self-report), and included only early stage colon cancer survivors. We examined associations between individual antihypertensive drug classes and colon cancer recurrence risk, several of which (CCBs and TDs) have not been examined previously. However, some of the sample sizes for individual drug classes were small, producing results with wide confidence intervals and requiring confirmation in additional studies. It is possible that our null results for antihypertensives are related to people using more than one type of antihypertensive medication where the hypothesized risk of recurrence goes in the opposite direction (for example, users of both ACEIs and TDs). Restricting our analyses to users of only one type of antihypertensive would have greatly limited the sample size for our analysis, since use of more than one type of antihypertensive is common in clinical practice [39]. Our study also has additional strengths, including use of longitudinal databases to ascertain many covariates. Our study sample was population-based, including colon cancer survivors from our tumor registries and not subject to participation bias. We also manually abstracted recurrence outcomes for study participants, which required substantial effort but produced high quality outcome data, which are not available from many population-based tumor registries.

A limitation of our study was sample size. The number of people diagnosed with early stage colon cancer who went on to have a recurrence or additional cancer was small despite including nearly 20 years of data from two health plans. This resulted in wide confidence intervals for our null results. It is possible that heterogeneity in development of different types of second primary cancers may be masking any association between CVD medications and second cancer events. However, we did not have sufficient numbers of different types of other cancers to examine these associations individually. Additional limitations of our analysis include some loss to follow-up among survivors; 16% of our population disenrolled from one of the health plans during follow-up, and we have no way to track recurrence outcomes for these people. There is also the potential for misclassification of exposure information for people who filled prescriptions but did not ingest the medication. Finally, we may have healthy user bias and/or unmeasured confounding (for example, due to lifestyle factors such as exercise) in our observational study. However, we attempted to account for this potential bias by adjusting for many confounders obtained via high quality medical record review and automated health plan data extraction.

## Conclusions

Statins and antihypertensives, two commonly used medications among older adults, were not associated with an increased or decreased risk of recurrence or any additional cancer event in a population-based sample of early-stage colon cancer survivors. Despite laboratory studies suggesting cellular mechanisms for CVD medications to either promote or inhibit cell growth, and subsequently increase or decrease cancer risk, we could not confirm these results in our study. This highlights the importance of conducting both laboratory and epidemiological studies because cellular mechanisms do not always translate directly into population-based studies. Given our results align with the null results found by previous studies, these should be reassuring to colon cancer patients, and their providers, who use these medications to manage comorbid cardiovascular disease, the leading cause of death in the United States [21, 40].

## Abbreviations

95%CI: 95% confidence interval
ACEI: Angiotensin-converting enzyme inhibitor
AJCC: American Joint Committee on Cancer
ARB: Angiotensin-II receptor blocker
BB: Beta blocker
CCB: Calcium channel blocker
HCSRN: Health Care System Research Network
HR: Hazard ratio
KPCO: Kaiser Permanente Colorado
KPWA: Kaiser Permanente Washington
RECORD: **Re**currence of **Co**lon cancer in **R**elation to **D**rug use
SEER: Surveillance Epidemiology and End Results
TD: Thiazide diuretic
VDW: Virtual Data Warehouse
